# Relationship between human exhalation diffusion and posture in face-to-face scenario with utterance

**DOI:** 10.1063/5.0038380

**Published:** 2021-02-23

**Authors:** Keiko Ishii, Yoshiko Ohno, Maiko Oikawa, Noriko Onishi

**Affiliations:** 1 Department of Mechanical Engineering, College of Science and Engineering, Aoyama Gakuin University, 5-10-1, Fuchnobe, Sagamihara 252-5258, Japan; 2 Yamano College of Aesthetics, 530, Yarimizu, Hachioji, Tokyo 192-0396, Japan

## Abstract

Because of the COVID-19, the world has been affected significantly. Not only health and medical problems but also the decline in life quality and economic activity due to the suspension of social activities cannot be disregarded. It is assumed that the virus is transmitted through coughing and sneezing; however, the possibility of airborne infection by aerosols containing viruses scattered in the air has become a popular topic recently. In airborne infections, the risk of infection increases when the mucous membrane is exposed to exhaled aerosols for a significant amount of time. Therefore, in this study, we visualize human breath using the smoke of electronic cigarettes as tracer particles. Exhalation when speaking was visualized for four human posture patterns. The result shows that the exhaled breath is affected by the body wall temperature; it rises when it remains in the boundary layer by wearing a mask. On the other hand, without a mask, it initially flows downward due to the structure of the nose and mouth, so it flows downward due to inertia and diffuses randomly. This finding is effective in reducing the risk of infection during face-to-face customer service.

## I. INTRODUCTION

Because of the COVID-19, many people are refraining from going out unnecessarily. To control the infection of the virus, contact with people must be controlled; however, this will result in significant economic downturn and life quality deterioration. A person has a higher risk of being infected when exposed to coughing or sneezing. However, it has been elucidated that the effects of airborne and aerosol infections due to breathing and speech cannot be disregarded.^1–9^ The higher the frequency of exposure to the eye, nose, and oral mucosa to aerosols containing pathogens, the higher is the risk of infection.^10,11^ In particular, because utterances are accompanied during customer service, long-term care, and medical practice, droplets larger than those generated during normal breathing may be scattered in the air, and the virus may become an aerosol and float in the air for a long time. Future investigations are required to elucidate the detailed infection mechanism from virological and medical perspectives. However, in reality, the risk of infection must be reduced before any activity is to be conducted. In particular, cosmetology, medical care, and long-term care are inevitable in human life; hence, it is difficult to refrain from activities associated with these areas or to use separators because contact is necessitated. An understanding of the spread of human breath spatially may result in a lower probability of aerosol exposure when providing customer and medical services, thereby reducing the risk of infection.

Bourouiba *et al.* and other researchers performed experimental visualizations, followed by hydrodynamic considerations.^12,13,14^ Because sneezing and coughing occur when the heightened pressure in the oral cavity is released, respiratory droplets are ejected over a wide area at high speed.^15^ Therefore, one should maintain a safe distance from a person who is sneezing or coughing. However, persons infected with the COVID-19 may be asymptomatic; hence, it is important to recognize the risk of having conversational contact with seemingly healthy humans. In a previous study, airflow visualization measurement experiments were conducted to understand infection control more effectively;^16–20^ however, most of them focused on sneezing and coughing. To verify the effects of wearing the mask, Verma visualized smoke diffusion by simulating exhalation based on a smoke generator and generated an airflow simulating cough from a mannequin wearing a mask and face shields.^21,22^ In the conventional experimental breath visualization, water vapor, smoke, etc., are used as tracer particles to simulate breath aerosols that diffuse through a mannequin; however, because breath is assumed to be affected by the human body temperature, the actual breath aerosol movement might differ by mannequin experiments. Melikov *et al.* used the thermal mannequins to investigate the diffusion of exhaled breath, considering the effects of body temperature.^23^ Experiments with mannequins ensure quantitativeness and reproducibility, and since toxic tracer particles can also be used, the gas concentration distribution in the space due to respiration has been obtained. On the other hand, to completely match the thermal characteristics of the mannequin with the human body, the system cost is high, and it is difficult to make the mannequin pose or speak arbitrarily. Many numerical and theoretical studies have been conducted to investigate the spread of exhaled breath, cough, and sneeze, where the temperature and humidity of the human body were considered,^24–27^ and actual exhaled breath diffusions were rarely measured based on experiments from the standpoint of infectious disease prevention. Exhalation has not been focused on, and scenes that assume face-to-face customer service have not been visualized and there are not many findings regarding the aerosol exposure in minimizing the disease transmission.^28,29^ Therefore, in this study, we assumed customer service in a beauty salon or long-term care to experimentally visualize the flow and diffusion of the actual exhaled breath of a person who is speaking. The exhaled breath released from the human body was experimentally visualized, and the results were considered from the viewpoint of thermal fluid.

## II. EXPERIMENT

Splashes of saliva containing virus are ∼1 *µ*m–1000 *µ*m, and the size of viruses such as the COVID-19 contained in them is ∼0.1 *µ*m.^30–33^ Respiratory droplets measuring ∼1000 *µ*m are primarily generated through coughing and sneezing, and general exhaled aerosols are ∼5 *µ*m or less. Therefore, in this experiment, electronic cigarettes were used as tracer particles. It has been reported that the liquid used in electronic cigarettes is primarily composed of glycerin and propylene glycol, and when heated, it becomes an aerosol with a particle size of 0.1 *µ*m–1 *µ*m.^34,35^ Therefore, if an electronic cigarette is used as a tracer particle, then the diffusion and advection behavior of the virus released by respiration from the human body can be experimentally simulated. VAPORESSO Veco One Plus (Tradeworks) electronic cigarettes were used. The electronic cigarette liquid (BI-SO) comprising 45% propylene glycol, 55% glycerin, and 5% fragrance was atomized by the electric cigarette, and the smokes were used as tracers. The experimental system comprised a pair of humans, laser (G3000, Kato Koken), and CMOS camera (FLIR GS3-U3-32S4). A laser sheet (532 nm) was incident on an arbitrary cross section, and light scattered from the aerosol of the electronic cigarette was captured by using a camera. Assuming that customer service was accompanied by actual utterances, the word “onegaishimasu” (Japanese greetings) was repeatedly spoken during the measurement. The exposure time of the camera was 20 ms, and the frame rate was 5 fps. The experiment was conducted at a beauty salon at Yamano College of Aesthetics in Hachioji, Tokyo, on September 30, 2020, at time 16:00–18:00 (temperature 23.0 °C–21.2 °C and humidity 57%–65%). After ventilating the room, the windows and doors were closed, no air-conditioning was turned on, and the experiment was conducted in a windless state. We analyzed the characteristics of the exhalation diffusion with and without a mask when a person was standing, sitting, facing down, or lying face-up. The mask was made of non-woven fabric. Because the airflow was turbulent, reproducibility was confirmed by capturing pictures three or more times in each case. The pose was captured by simulating customer service at the beauty salon. A significant amount of similar face-to-face contact would occur not only in cosmetology but also in long-term and medical care.

## III. RESULTS AND DISCUSSIONS

**FIG. 1.**
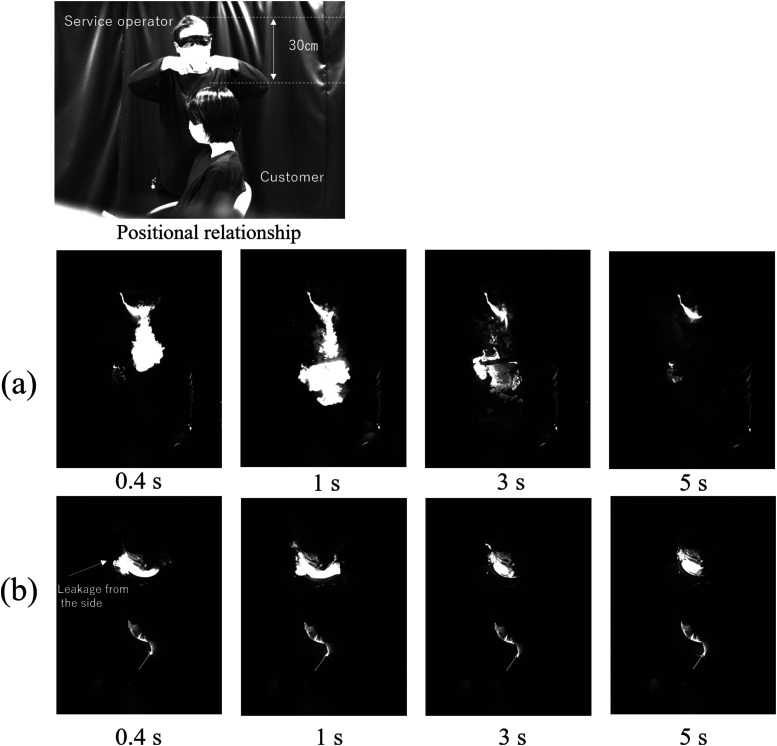
Front images of exhaled breath of a standing person: (a) without a mask and (b) with a mask. Multimedia views: https://doi.org/10.1063/5.0038380.110.1063/5.0038380.1; https://doi.org/10.1063/5.0038380.210.1063/5.0038380.2

First, the exhaled breath with utterance of a standing person is discussed. Hereafter, the “exhaled breaths” of all the experimental results of this study were accompanied with utterance. The image at the top of Fig. 1 shows the positional relationship of persons assuming that a beautician performs treatments such as haircuts from the side of the customer. Figures 1(a) and 1(b) show the visualization results of the service operator’s exhaled breath with and without a mask, respectively, while maintaining this positional relationship. The laser sheet was incident parallel to the position 10 cm in front of the service operator’s body. As shown in Fig. 1(a), the exhaled breath was successfully visualized by scattering the laser beam from the aerosols produced by the electronic cigarette. The exhaled aerosols diffused downward in a jet shape and then horizontally. In cosmetology and long-term care, the customer is often positioned lower than the service operator; therefore, it is noteworthy that the exhaled breath moves downward. This downward flow occurred because the breath released downward initially due to the structure of the nose and mouth, and subsequently flowed due to inertia. Meanwhile, as shown in Fig. 1(b), the exhaled breath barely flowed in front of the service operator, i.e., the laser sheet position. This is because the aerosols flowed along the gap between the face surface and the mask. Because the exhaled breath flowing downward in the front can be suppressed significantly, the service operator can reduce the risk of infection to the customer by wearing a mask. Due to the visibility of the exhaled breath, the customer was removed from the seat in the image of Fig. 1(a). However, even if there was a customer, it was confirmed that the exhaled jet goes to the customer as shown in this result. The results of side observations of the case where the customer was in front of the service operator were explained in the following experimental results.

**FIG. 2.**
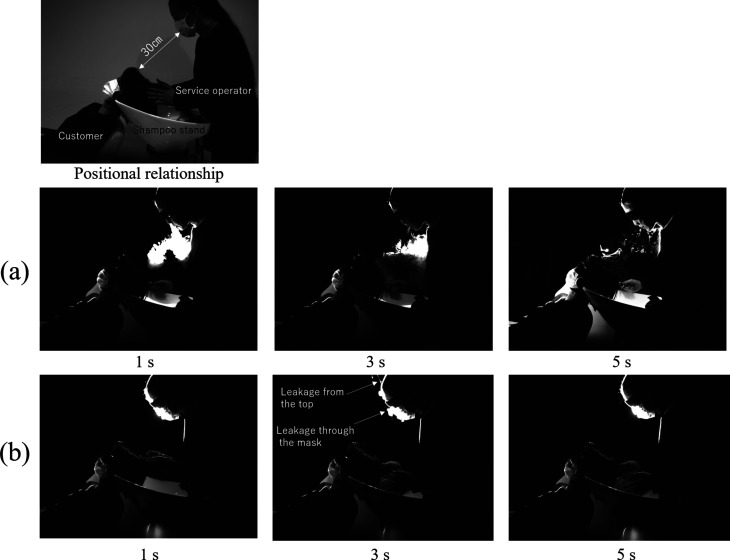
Side images of exhaled breath of a standing person: (a) without a mask and (b) with a mask. Multimedia views: https://doi.org/10.1063/5.0038380.310.1063/5.0038380.3; https://doi.org/10.1063/5.0038380.410.1063/5.0038380.4

Next, by observing the exhaled breath of the standing service operator from the side, the three-dimensional spread of exhaled breath is discussed. The image at the top of Fig. 2 shows the positional relationship assuming that the beautician performs shampoo treatments from behind the customer. This is a general positional relationship in shampoo treatments performed in beauty and long-term care. The customer sat and rested her head on the shampoo stand. The laser sheet was positioned vertically through the center of the face of the service operator and the customer. As shown in Fig. 2(a), the practitioner’s exhaled breath moved downward, similar to the observation at the front. Subsequently, the aerosols stagnated in the space between the customer and the shampoo stand. Meanwhile, when a mask was worn, as shown in Fig. 2(b), exhalation jet did not occur in front of the service operator. A slight leakage of aerosol from the mask was observed; however, it did not spread forward or downward. It is noteworthy that although exhaled air was observed above the mask, almost no exhaled air was observed from the vicinity of the chin. This is because the fit is much tighter at the chin compared to large gaps that exist between the nose bridge and the mask. Therefore, all of the leakage occurs through the top of the mask.

**FIG. 3.**
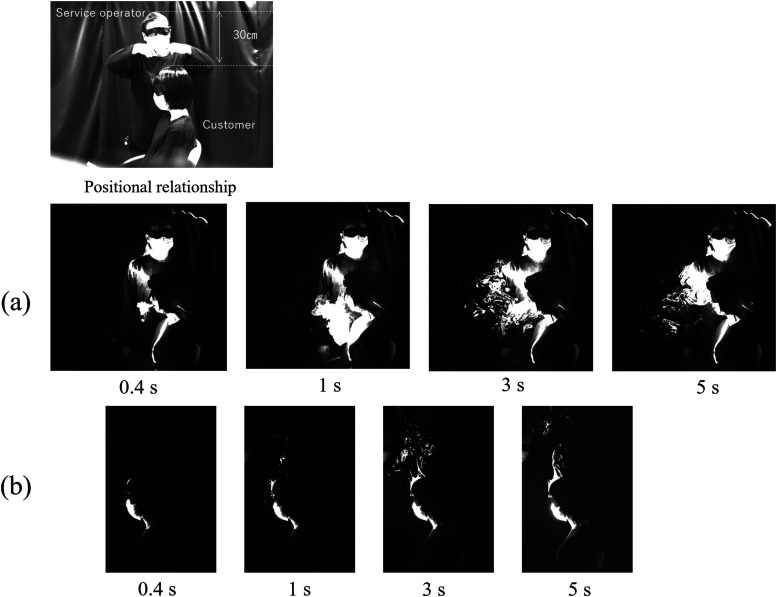
Side image of exhalation of a sitting person: (a) without mask and (b) with mask. Multimedia views: https://doi.org/10.1063/5.0038380.510.1063/5.0038380.5; https://doi.org/10.1063/5.0038380.610.1063/5.0038380.6

Next, the exhalation of the seated person is discussed. Figure 3 shows the visualization results of the positional relationship when the beautician cuts the hair from the side or front of the customer. The height difference between the two persons was 30 cm. Figure 3(a) shows the visualization result of the exhaled breath of a sitting customer not wearing a mask. Initially, the customer’s exhaled breath moved downward, similar to the exhalation when standing. Subsequently, the exhaled breath remained in front of the human body for a significant amount of time and gradually increased from 3 s to 5 s. This is because the aerosols that reattached near the surface of the human body tended to warm up and then rise. Hence, in front of a sitting person than a standing person, the exhaled breath rises gradually, which may increase the risk of airborne infection in the vicinity. Figure 3(b) shows the results when the sitting customer wore a mask. Because it was difficult to distinguish between light scattered from the mask of the practitioner behind the customer and the exhaled breath, the result of changing the position of the service operator is presented instead. The mask completely suppressed the forward expiratory jet, whereas the exhaled breath leaked from the upper part of the mask. This exhaled air flowed along the head, rose, and then released from the crown of the head. This occurred because the temperature near the human body was higher than the air temperature, causing a flow that rose along the surface of the human body. Therefore, the area near the crown of the head was a region with a high risk of infection. In addition, in a ventilation environment where multiple ventilation fans were placed on the ceiling of the room, thereby causing constant updrafts, it was assumed that the exhaled aerosols can be effectively released to the outside without contacting others.

**FIG. 4.**
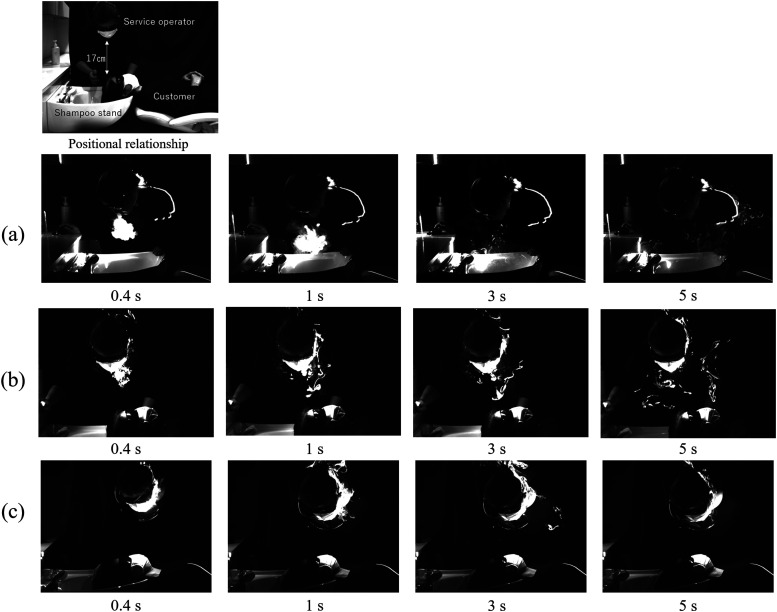
Side image of exhalation of a sitting person: (a) without a mask and (b) with mask; (c) with a mask and face shield. Multimedia views: https://doi.org/10.1063/5.0038380.710.1063/5.0038380.7; https://doi.org/10.1063/5.0038380.810.1063/5.0038380.8

Figure 4 shows the visualization result of exhaled breath when the treatment was performed facing down. The shortest distance between the service operator and the customer was 17 cm. The positional relationship between the two persons was assumed as that when shampooing was performed from the customer’s side in a beauty salon. The customer was lying on a flat-type shampoo stand. The laser sheet passed through the practitioner’s mouth and was projected perpendicular to the face. Figure 4(a) shows the breath of the practitioner not wearing a mask. For infection control, the customer was not seated at the seat when these data were obtained. As the before results, the exhaled breath flowed in the direction of gravity when the mask was not worn. Subsequently, it remained in the shampoo stand and then diffused into the space. Figure 4(b) shows the exhalation of a service operator wearing a mask. Unlike the standing and sitting positions, the exhalation leaked from the mask and fell in the direction of gravity. When facing down, the aerosols leaking from the mask left the temperature boundary layer of the human body and moved downward due to the structure of the nose and mouth. The temperature boundary layer generated in a substance whose temperature is higher than that of the surrounding atmosphere is considered to be thin at the lower part of the object, whereas the exhaled air leaking from the lower part is considered to be easily separated from the human body. Subsequently, the exhaled air diffused into the space in a complicated manner while interfering with the customer. The diffusion of exhaled breath would be more complicated during an actual shampooing service involving a hot shower. Figure 4(c) shows the breath of a service operator wearing a face shield in addition to a mask. When the face shield was used, the exhaled air flowed backward along the temporal region of the head and did not reach the customer. The face shield redirected the exhaled breath and promoted the reattachment to the body. Consequently, the face shield promoted the rise of the exhaled breath. Hence, it is more effective to wear both a mask and a face shield when providing services to customers.

**FIG. 5.**
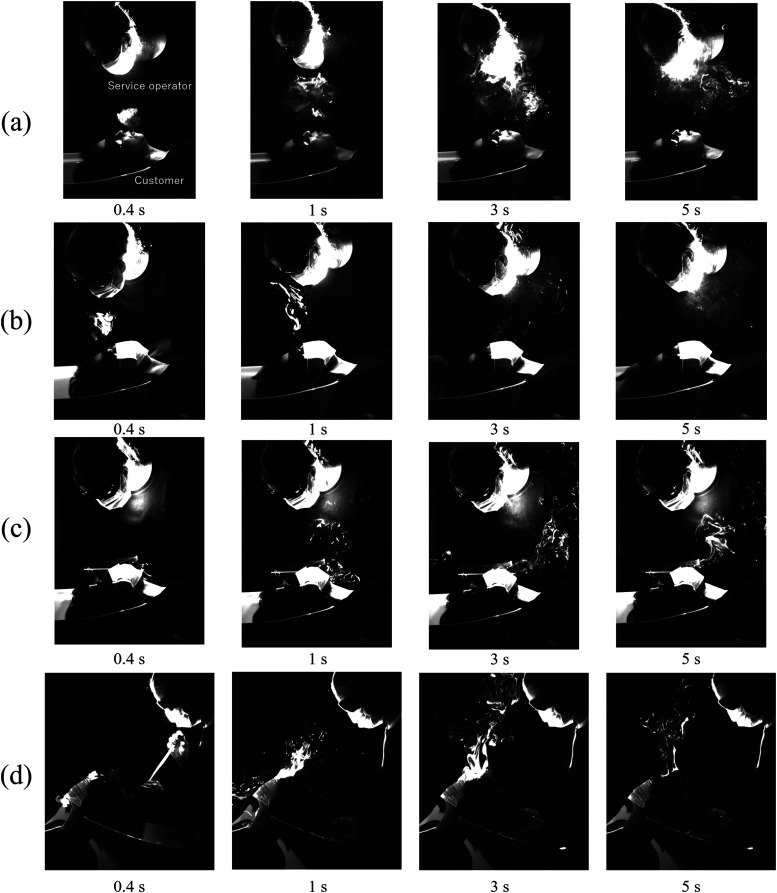
Images of exhalation of a lying and facing-up person: (a) without a mask; (b) with a mask; (c) with a mask and face shield; and (d) with a mask and facing diagonally upward. Multimedia views: https://doi.org/10.1063/5.0038380.910.1063/5.0038380.9; https://doi.org/10.1063/5.0038380.1010.1063/5.0038380.10

Finally, the characteristics of exhaled breath when facing upward are discussed. Figure 5(a) shows the exhalation when the customer lying on the shampoo stand was not wearing a mask. The exhaled breath of the customer facing upward was turbulent and diffused upward. Figure 5(b) shows the case where a mask was worn. Exhaled breath leaking from the top of the mask was confirmed from 0.4 s to 1 s. Subsequently, the exhaled air diffused between the two persons in a complicated manner while interfering with the exhaled air transmitted from the mask. The non-woven mask did not allow much of the aerosols of the electronic cigarette to permeate; however, the breath permeated, and the aerosols leaking from the vicinity of the nose were agitated by the permeated breath. Hence, the risk of infection at the region directly above the customers facing upwards was considered to be high. Meanwhile, as shown in Fig. 5(d), when wearing a mask and facing diagonally upward, the aerosols that leaked from the top did not interfere with the permeated breath and tended to move upward in a less turbulent manner. However, it is noteworthy that if a person is present at the back, then the exhalation tends to move to the back. Figure 5(c) shows a customer facing up who is wearing a mask and a face shield. Exhaled air rose from the edge of the face shield and diffused into the space. Although a direct hit of the exhaled breath to the service operator directly above can be avoided, when facing upward, the exhaled breath rose turbulently in all cases. Because patients are often lying down in medical and long-term care settings, it is noteworthy that the exhaled jet rises near the head during contact.

## IV. CONCLUSION

We visualized the actual human breath to obtain guidelines for social activities such as customer service while suppressing the risk of infection. The following conclusions were obtained:

• When not wearing a mask, the shape of the nose and mouth structure made exhaled air move downward due to inertia.
• By wearing a mask, when standing or sitting, the exhaled breath tends to reattach to the human body, and the aerosols flowed up along the body.
• When facing downward, the aerosols tended to separate from the temperature boundary layer of the human body. The exhaled air that has passed through the mask tends to move downward under the influence of the initial expiratory jet generated by the nose and mouth. Hence, it is effective to wear a face shield when liaising with a person below.
• When facing upward, the aerosols leaking from the upper part of the mask interfered with the exhaled air transmitted from the mask surface; furthermore, the aerosols were agitated and flowed in a complicated manner even when the mask was worn. Because the exhaled air flowed to the back, care must be established when contacting from behind.
• By wearing a non-woven mask, the injection of aerosols to the front was effectively suppressed.

Hence, in this study, we focused on the expiratory characteristics affected by the human body temperature and posture; additionally, we measured the flow field based on those characteristics.

## DATA AVAILABILITY

The data that support the findings of this study are available from the corresponding author upon reasonable request.

## References

[1] J. T. Brooks, J. C. Butler, and R. R. Redfield, “Universal masking to prevent SARS-CoV-2 transmission-the time is now,” JAMA: J. Am. Med. Assoc. 324, 635 (2020). 10.1001/jama.2020.13107 PMC8607819 32663243

[2] D. K. Milton, M. P. Fabian, B. J. Cowling, M. L. Grantham, and J. J. McDevitt, “Influenza virus aerosols in human exhaled breath: Particle size, culturability, and effect of surgical masks,” PLoS Pathog. 9, e1003205 (2013). 10.1371/journal.ppat.1003205 23505369 PMC3591312

[3] J. Gralton, E. R. Tovey, M.-L. Mclaws, and W. D. Rawlinson, “Respiratory virus RNA is detectable in airborne and droplet particles,” J. Med. Virol. 85, 2151 (2013). 10.1002/jmv.23698 23959825

[4] W. E. Bischoff, K. Swett, I. Leng, and T. R. Peters, “Exposure to influenza virus aerosols during routine patient care,” J. Infect. Dis. 207, 1037 (2013). 10.1093/infdis/jis773 23372182

[5] P. Bahl, C. Doolan, C. de Silva, A. A. Chughtai, L. Bourouiba, and C. R. MacIntyre, “Airborne or droplet precautions for health workers treating COVID-19?,” J. Infect. Dis. 189, jiaa189 (2020). 10.1093/infdis/jiaa189 PMC7184471 32301491

[6] Y. Liu, Z. Ning, Y. Chen, M. Guo, Y. Liu, N. K. Gali, L. Sun, Y. Duan, J. Cai, D. Westerdahl, X. Liu, K. Xu, K.-f. Ho, H. Kan, Q. Fu, and K. Lan, “Aerodynamic analysis of SARS-CoV-2 in two Wuhan hospitals,” Nature 582, 557 (2020). 10.1038/s41586-020-2271-3 32340022

[7] M. Abuhegazy, K. Talaat, O. Anderoglu, S. V. Poroseva, and K. Talaat, “Numerical investigation of aerosol transport in a classroom with relevance to COVID-19,” Phys. Fluids 32, 103311 (2020). 10.1063/5.0029118 PMC7583363 33100808

[8] J. Xi, X. A. Si, and R. Nagarajan, “Effects of mask-wearing on the inhalability and deposition of airborne SARS-CoV-2 aerosols in human upper airway,” Phys. Fluids 32, 123312 (2020). 10.1063/5.0034580 PMC7757581 33362401

[9] G. A. Somsen, C. J. M. van Rijn, S. Kooij, R. A. Bem, and D. Bonn, “Measurement of small droplet aerosol concentrations in public spaces using handheld particle counters,” Phys. Fluids 32, 121707 (2020). 10.1063/5.0035701 PMC7757574 33362399

[10] S. A. Lauer, K. H. Grantz, Q. Bi, F. K. Jones, Q. Zheng, H. R. Meredith, A. S. Azman, N. G. Reich, and J. Lessler, “The incubation period of coronavirus disease 2019 (COVID-19) from publicly reported confirmed cases: Estimation and application,” Ann. Int. Med. 172, 577 (2020). 10.7326/m20-0504 32150748 PMC7081172

[11] C. R. MacIntyre and A. A. Chughtai, “A rapid systematic review of the efficacy of face masks and respirators against coronaviruses and other respiratory transmissible viruses for the community, healthcare workers and sick patients,” Int. J. Nurs. Stud. 108, 103629 (2020). 10.1016/j.ijnurstu.2020.103629 32512240 PMC7191274

[12] B. E. Scharfman, A. H. Techet, J. W. M. Bush, and L. Bourouiba, “Visualization of sneeze ejecta: Steps of fluid fragmentation leading to respiratory droplets,” Exp. Fluids 57, 24 (2016). 10.1007/s00348-015-2078-4 32214638 PMC7088075

[13] L. Bourouiba, E. Dehandschoewercker, and J. W. M. Bush, “Violent expiratory events: On coughing and sneezing,” J. Fluid Mech. 745, 537 (2014). 10.1017/jfm.2014.88

[14] L. Bourouiba, “Turbulent gas clouds and respiratory pathogen emissions: Potential Implications for Reducing transmission of COVID-19,” JAMA: J. Am. Med. Assoc. 323, 1837 (2020). 10.1001/jama.2020.4756 32215590

[15] G. Busco, S. R. Yang, J. Seo, and Y. A. Hassan, “Sneezing and asymptomatic virus transmission,” Phys. Fluids 32, 073309 (2020). 10.1063/5.0019090 PMC7367211 32684746

[16] W. G. Lindsley, F. M. Blachere, B. F. Law, D. H. Beezhold, and J. D. Noti, “Efficacy of face masks, neck gaiters and face shields for reducing the expulsion of simulated cough-generated aerosols,” Aerosol Sci Tech (published online, 2021). 10.1080/02786826.2020.1862409 PMC9345365 35924077

[17] J. W. Tang, T. J. Liebner, B. A. Craven, and G. S. Settles, “A schlieren optical study of the human cough with and without wearing masks for aerosol infection control,” J. R. Soc. Interface 6, S727 (2009). 10.1098/rsif.2009.0295.focus 19815575 PMC2843945

[18] H. Nishimura, S. Sakata, and A. Kaga, “A new methodology for studying dynamics of aerosol particles in sneeze and cough using a digital high-vision, high-speed video system and vector analyses,” PLoS One 8, e80244 (2013). 10.1371/journal.pone.0080244 24312206 PMC3842286

[19] S. Zhu, S. Kato, and J.-H. Yang, “Study on transport characteristics of saliva droplets produced by coughing in a calm indoor environment,” Build. Environ. 41, 1691 (2006). 10.1016/j.buildenv.2005.06.024

[20] H. Wang, Z. Li, X. Zhang, L. Zhu, Y. Liu, and S. Wang, “The motion of respiratory droplets produced by coughing,” Phys. Fluids 32, 125102 (2020). 10.1063/5.0033849 PMC7757605 33362402

[21] S. Verma, M. Dhanak, and J. Frankenfield, “Visualizing the effectiveness of face masks in obstructing respiratory jets,” Phys. Fluids 32, 061708 (2020). 10.1063/5.0016018 PMC7327717 32624649

[22] S. Verma, M. Dhanak, and J. Frankenfield, “Visualizing droplet dispersal for face shields and masks with exhalation valves,” Phys. Fluids 32, 091701 (2020). 10.1063/5.0022968 PMC7497716 32952381

[23] A. Melikov and J. Kaczmarczyk, “Measurement and prediction of indoor air quality using a breathing thermal manikin,” Indoor Air 17, 50 (2007). 10.1111/j.1600-0668.2006.00451.x 17257152

[24] T. Dbouk and D. Drikakis, “On coughing and airborne droplet transmission to humans,” Phys. Fluids 32, 053310 (2020). 10.1063/5.0011960 PMC7239332 32574229

[25] S. Chaudhuri, S. Basu, P. Kabi, V. R. Unni, and A. Saha, “Modeling the role of respiratory droplets in COVID-19 type pandemics,” Phys. Fluids 32, 063309 (2020). 10.1063/5.0015984 PMC7327718 32624650

[26] T. Dbouk and D. Drikakis, “On respiratory droplets and face masks,” Phys. Fluids 32, 063303 (2020). 10.1063/5.0015044 PMC7301882 32574231

[27] F. Akagi, I. Haraga, S.-i. Inage, and K. Akiyoshi, “Effect of sneezing on the flow around a face shield,” Phys. Fluids 32, 127105 (2020). 10.1063/5.0031150 PMC7757660 33362403

[28] S. Feng, C. Shen, N. Xia, W. Song, M. Fan, and B. J. Cowling, “Rational use of face masks in the COVID-19 pandemic,” Lancet Respir. Med. 8, 434 (2020). 10.1016/s2213-2600(20)30134-x 32203710 PMC7118603

[29] J. Xiao, E. Y. C. Shiu, H. Gao, J. Y. Wong, M. W. Fong, S. Ryu, and B. J. Cowling, “Nonpharmaceutical measures for pandemic influenza in nonhealthcare settings-personal protective and environmental measures,” Emerg. Infect. Dis. 26, 967 (2020). 10.3201/eid2605.190994 32027586 PMC7181938

[30] S. Yang, G. W. M. Lee, C.-M. Chen, C.-C. Wu, and K.-P. Yu, “The size and concentration of droplets generated by coughing in human subjects,” J. Aerosol Med. 20, 484 (2007). 10.1089/jam.2007.0610 18158720

[31] C. Y. H. Chao, M. P. Wan, L. Morawska, G. R. Johnson, Z. D. Ristovski, M. Hargreaves, K. Mengersen, S. Corbett, Y. Li, X. Xie, and D. Katoshevski, “Characterization of expiration air jets and droplet size distributions immediately at the mouth opening,” J. Aerosol Sci. 40, 122 (2009). 10.1016/j.jaerosci.2008.10.003 32287373 PMC7126899

[32] X. Xie, Y. Li, H. Sun, and L. Liu, “Exhaled droplets due to talking and coughing,” J. R. Soc. Interface 6, S703 (2009). 10.1098/rsif.2009.0388.focus 19812073 PMC2843952

[33] Z. Y. Han, W. G. Weng, and Q. Y. Huang, “Characterizations of particle size distribution of the droplets exhaled by sneeze,” J. R. Soc. Interface 10, 20130560 (2013). 10.1098/rsif.2013.0560 24026469 PMC3785820

[34] M. J. Oldham, J. Zhang, M. J. Rusyniak, D. B. Kane, and W. P. Gardner, “Particle size distribution of selected electronic nicotine delivery system products,” Food Chem. Toxicol. 113, 236 (2018). 10.1016/j.fct.2018.01.045 29408542

[35] M. Belka, F. Lizal, J. Jedelsky, M. Jicha, and J. Pospisil, “Measurement of an electronic cigarette aerosol size distribution during a puff,” EPJ Web Conf. 143, 02006 (2017). 10.1051/epjconf/201714302006

